# Ultrasound-guided acupotomy release of A1 pulley in the treatment of trigger thumb: A case report

**DOI:** 10.1097/MD.0000000000042877

**Published:** 2025-06-13

**Authors:** Jingxi Ou, Chengcheng Liu, Li Liu, Pengyan Han

**Affiliations:** a Chongqing Banan District Hospital of Traditional Chinese Medicine, Chongqing, China.

**Keywords:** A1 pulley, acupotomy, case report, trigger thumb, ultrasound-guidance

## Abstract

**Rationale:**

Trigger thumb, characterized by A1 pulley thickening secondary to tendon sheath stenosis, represents a common clinical entity. While multiple therapeutic options exist, ultrasound-guided acupotomy has gained significant clinical adoption in China yet remains inadequately documented in the scientific literature. This study reports a case of trigger thumb managed with ultrasound-guided acupotomy.

**Patient concerns:**

A 49-year-old female presented with a 3-month history of audible clicking and discomfort during flexion-extension of the left thumb. Physical examination revealed pain, swelling, and persistent tenderness localized to the base and palmar aspect of the affected thumb, exacerbated by routine manual activities. No antecedent trauma was reported. A significant reduction in the left thumb’s range of motion was objectively documented.

**Diagnoses:**

The patient was diagnosed with trigger thumb (stenosing tenosynovitis) based on characteristic clinical manifestations and physical findings.

**Interventions:**

Ultrasound-guided acupotomy was performed to release the stenosed A1 pulley of the left thumb.

**Outcomes:**

Postintervention outcomes included immediate pain resolution, complete cessation of clicking sounds, significant restoration of thumb range of motion, and functional recovery of the affected digit.

**Lessons:**

This case demonstrates that ultrasound-guided acupotomy achieves rapid and substantial therapeutic efficacy in trigger thumb management. The technique preserves tendon integrity while minimizing procedural risks, establishing its potential as a valuable clinical intervention warranting broader investigation and application.

## 1. Introduction

Thumb stenosing tenosynovitis, commonly referred to as “trigger thumb,” is a noninfectious inflammatory condition arising from excessive friction between the tendon and its sheath due to strain-induced injury of the thumb flexor tendon,^[1–3]^ This disorder predominantly affects middle-aged and elderly women, particularly those aged 50 to 60 years, with a male-to-female incidence ratio of approximately 1:7. The prevalence in the general population is 2.6%, while it increases to 10% among diabetic individuals.^[4]^ Additionally, the thumb and ring finger are more commonly affected compared to the middle, index, or little fingers.^[5]^ In the early stages, patients often experience pain, stiffness, and other discomforts, which may be alleviated by activity.^[6]^ As the condition progresses, the enlarged tendon encounters difficulty passing through the narrowed tendon sheath, leading to painful symptoms. In severe cases, this can result in tendon constriction, locking, and even an inability to flex the finger, significantly impacting the patient’s daily activities and quality of life.^[2]^

The treatment principles for trigger thumb emphasize pain relief, functional restoration, and localized interventions. Current management of symptomatic trigger thumb includes nonsurgical therapies, physical therapy, and local corticosteroid injections, with efficacy rates of 45% to 80%.^[7]^ Corticosteroid injections, widely used in clinical practice, primarily involve involve intra-sheath injection and peri-pulley injection.

In the intra-sheath injection approach, the needle is advanced into the space between the inner surface of the A1 pulley and the palmar aspect of the thumb flexor tendon for synovial sheath administration. The peri-pulley injection positions the needle in the space between subcutaneous fat tissue and the palmar surface of the A1 pulley for parapulley compartment injection. Both methods reduce inflammation, alleviate pain, and restore tendon function.^[8]^ Ultrasound-guided corticosteroid injection enhances precision, minimizing iatrogenic injury to flexor tendons, adjacent pulleys, and digital neurovascular bundles.^[9]^ However, studies show no significant difference in long-term outcomes (1-year follow-up) between intra-sheath injection and peri-pulley injection,^[10]^ with both limited by incomplete resolution of underlying tendon compression.

For refractory cases, open surgical release is indicated. This technique visualizes the A1 pulley, enabling complete decompression of the stenotic tendon sheath.^[11]^ Complications include infection, weakened grip strength, nerve injury, joint stiffness, flexor tendon bowstringing, and scar formation, with reported rates up to 12%.^[12]^

The Percutaneous Release Technique serves as an alternative to open surgical release. Although this procedure does not provide full visualization of the A1 pulley during operation – potentially leading to an elevated risk of nerve injury and incomplete release of thickened, adherent pulley tissue – it demonstrates advantages in clinical outcomes, including reduced postoperative pain duration and accelerated recovery of hand function.^[13]^

The ultrasound-guided percutaneous A1 pulley release technique employs a hypodermic needle for pulley release, demonstrating lower complication rates than open surgery, though risks persist.^[14]^

Acupotome is a minimally invasive therapeutic instrument that integrates the features of an acupuncture needle and a surgical scalpel into a single device. It offers dual advantages in incising and separating abnormal scar tissue and contracted regions. By combining the benefits of both acupuncture and surgical techniques, acupotomy provides both therapeutic and operative effects. Traditional needle-knife therapy predominantly depends on the clinician’s experience and lacks precise imaging guidance, which can compromise treatment efficacy and safety. The advancement of ultrasound visualization technology has significantly enhanced the safety and effectiveness of acupotomy treatments. With ultrasound-guidance, clinicians can clearly visualize the morphology and position of the tendon sheath, tendon, and surrounding structures, enabling accurate localization and operation, thereby improving treatment outcomes.^[4]^ Recent studies highlight the efficacy of Ultrasound-guided acupotomy release, which anatomically avoids neurovascular and tendon damage, proving safer than ultrasound-guided percutaneous A1 pulley release.^[15]^

In this paper, we present a case of ultrasound-guided acupotomy release of the A1 pulley for the treatment of trigger thumb. We also evaluate the application and efficacy of ultrasound-guided acupotomy in managing thumb stenosing tenosynovitis.

## 2. Description

All procedures in this study were carried out in strict adherence to the ethical standards set by institutional and/or national research ethics committees, as well as the guidelines outlined in the 2024 revision of the Declaration of Helsinki. Following comprehensive communication and detailed discussion, the patient consented to undergo ultrasound-guided acupotomy. Written informed consent for the publication of this article and any accompanying images was obtained from the patient. The editorial office of this journal has access to a copy of the signed consent form for review.

## 3. Case presentation

A 49-year-old female patient presented to the Acupuncture and Moxibustion Outpatient Department at Chongqing Banan District Hospital of Traditional Chinese Medicine, reporting a 3-month history of significant pain in the first metacarpophalangeal joint of her left thumb. Over the past week, she experienced a progressive worsening of symptoms. Two months prior, she consulted an orthopedic specialist who prescribed nonsteroidal anti-inflammatory drugs and topical corticosteroid injections. While the pain had slightly improved, other symptoms persisted. The orthopedist recommended surgical intervention, which the patient declined due to concerns about surgery. Currently, she relies exclusively on her right hand for grasping activities and avoids using her left hand. Within the month preceding this visit, she had not undergone any systemic treatments such as local block injections or surgical procedures. The patient has no history of hyperuricemia, diabetes, or related conditions. Blood tests conducted at other hospitals revealed a negative Rh factor and normal uric acid levels, ruling out rheumatic diseases and gouty arthritis.

**Figure 1.**
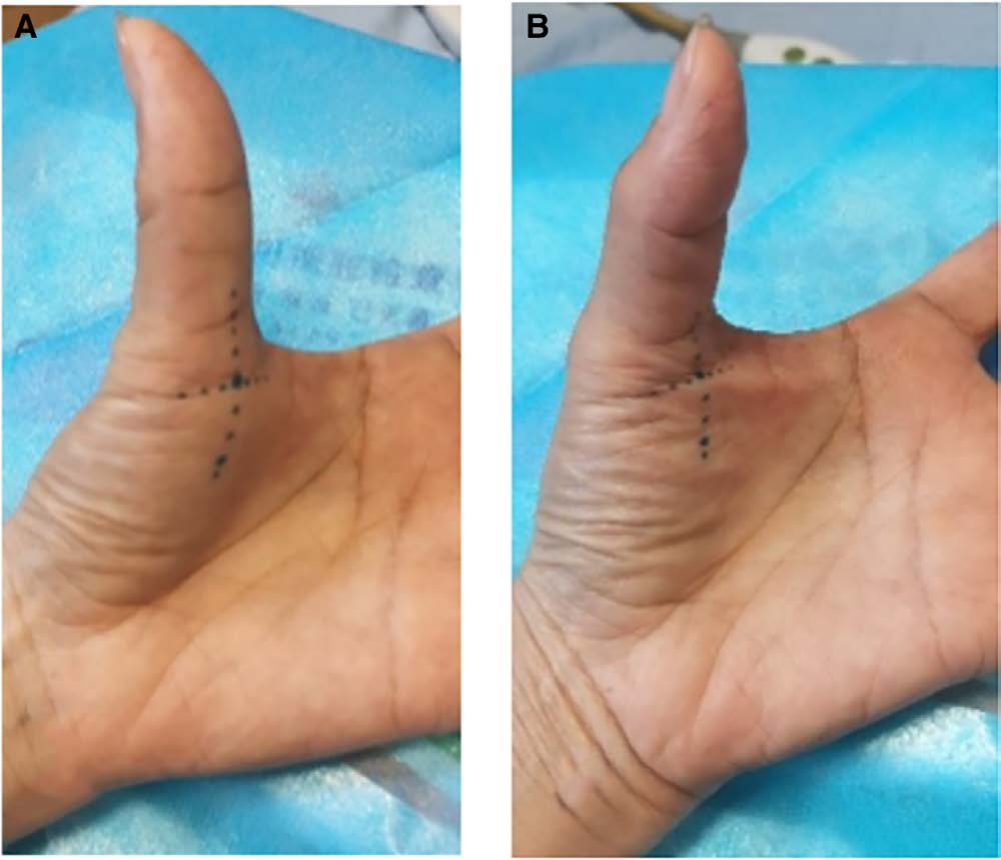
(A) Examination before treatment: Full extension; (B) pretreatment flexion state: ROM of the first interphalangeal joint = 5°.

Upon clinical evaluation, the patient reported that gripping activities intensified her pain, whereas periods of rest afforded some relief. The patient exhibited difficulty in flexing the metacarpophalangeal joint of her affected thumb on the examination table. Palpation revealed significant tenderness at the 1st metacarpophalangeal joint of the thumb, along with a fixed and hard nodule palpable on the medial aspect of the joint. Examination findings indicated that the range of flexion motion for the affected thumb’s metacarpophalangeal joint was limited to 0° to 5°, compared to the healthy thumb’s range of 0° to 90° (Fig. 1A, B). The patient’s Visual Analog Scale (VAS) pain score was 8.

The Quinnell classification was grade IV. Based on the patient’s history, clinical manifestations, and physical examination findings, along with necessary imaging studies (color Doppler ultrasound), a diagnosis of stenosing tenosynovitis of the flexor pollicis longus tendon was confirmed.

## 4. Inspection

### 4.1. Inspection method

**Figure 2.**
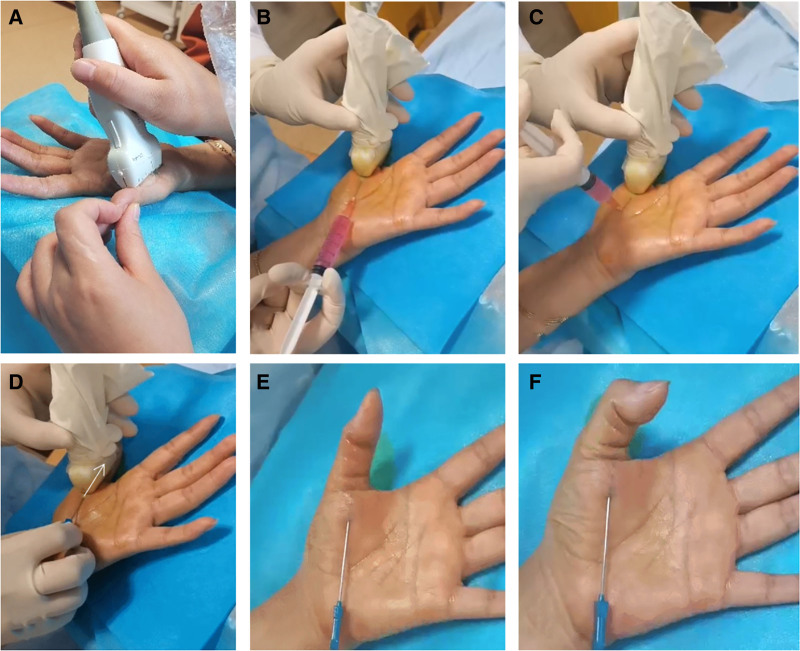
(A) Location of ultrasound examination; (B, C) local injection of infiltration anesthesia; (D) ultrasound-guided acupotomy longitudinal cutting; (E, F) flexion and extension of the left thumb after acupotomy treatment.

The operator guides the patient into a comfortable seated position with the left elbow slightly flexed (between 60° and 90°) and resting relaxed on the examination table, with the left hand naturally placed, palm up, and fingers fully spread and moderately spaced. For thumb evaluation, the forearm rotation angle is slightly adjusted to ensure that the dorsal surface of the thumb lightly contacts the bed surface, optimizing the inspection field of view. A coupling agent is applied to the metacarpophalangeal joint area of the affected finger to enhance ultrasonic conduction. A high-frequency ultrasound probe is used to make close contact with the skin, employing both longitudinal and transverse scanning techniques to display the long-axis structure and cross-sectional morphology of the tendon (Fig. 2A). Color Doppler ultrasound is utilized for precise hemodynamic assessment.

### 4.2. Inspection contents

(1) Under ultrasonic monitoring, the tendinopathy site was evaluated in detail, with the metacarpophalangeal joint area scanned using both longitudinal and transverse sections. The following signs were specifically assessed:
  ① Abnormal tendon morphology, characterized by localized enlargement, presenting a flattened, gourd-like shape with blurred margins and limited mobility.
  ② Hyperemia and edema of the tendon sheath tissue, resulting in increased thickness and reduced echogenicity.
  ③ Increased fluid dark areas within the synovial sheath, indicative of exudate accumulation.
  ④ Detection of hyperechoic spots or foci at the margins of the tendon sheath.
  ⑤ Enhanced local blood flow signals.
(2) Ultrasonic Evaluation of Tendon Sheath, Tendon, and Additional Parameters.

Once the long-axis and cross-sectional views were clearly visualized in the ultrasound images, the images were stabilized for accurate measurement. Key anatomical parameters were determined using the ultrasound system’s measurement tools and surface reference markers.

**Figure 3.**
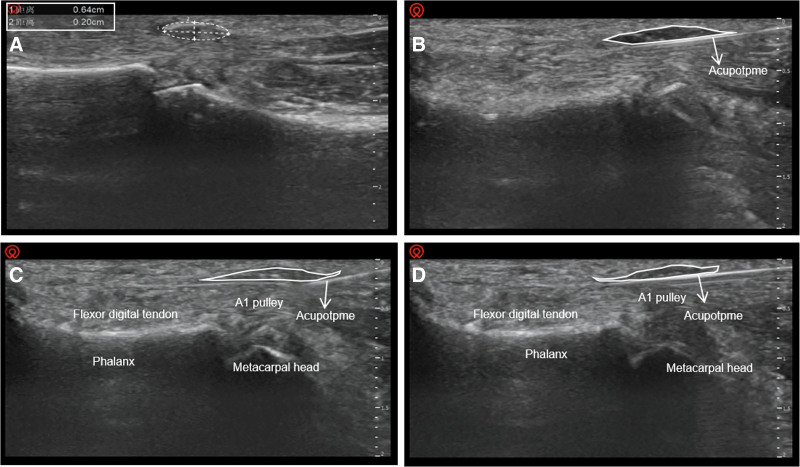
(A) The thickness of A1 pulley slip measured by ultrasound before treatment was 0.64 cm × 0.20 cm; (B) infiltration anesthesia with local injection under ultrasound; (C, D) ultrasound-guidance acupotome cutting path.

In the longitudinal view, the thickness of the A1 pulley on the affected side was compared with that on the unaffected side. The thickening and irregular shape of the tendons adjacent to the A1 pulley due to pathology were evaluated, allowing for quantification of the thickness differences between the flexor tendons bilaterally (Fig. 3A). Additionally, the projection of the affected finger’s A1 pulley on the body surface was marked and measured. The linear distance from the proximal to the distal end of the palmar transverse line was calculated to establish the spatial relationship of the lesion.

Switching to the transverse section, the focus shifted to measuring the distance between the centerline of the A1 pulley and the digital arteries on both sides. Upon completing these measurements, the insertion point for Acupotome was clearly indicated on the body surface.

## 5. Treatment

Following the completion of the ultrasound examination and anatomical positioning, the patient’s palm was positioned on the operating table with the center of the palm facing upward and the metacarpophalangeal joint hyperextended to 20°. The operator first identified the midpoint of the A1 pulley of the affected thumb, then extended 0.5 cm along the midline of the tendon towards the palm to determine the puncture starting point. Extensive disinfection using iodine was performed to ensure that the disinfection area covered approximately 2 to 3 cm above the wrist crease.

For the treatment of area A1, a precisely prepared 5 mL anti-inflammatory and analgesic solution (comprising 2 mL of 2% lidocaine, 1 mL of vitamin B_12_, 0.5 mL of betamethasone, and 1.5 mL of 0.9% saline) was used for meticulous layered infiltration anesthesia (Figs. 2B, C and 3B).

**Figure 4.**
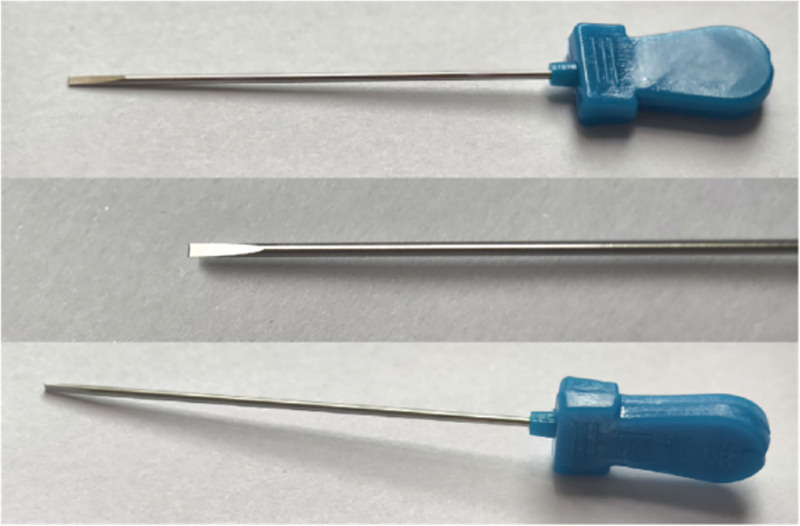
Acupotome specification (1.0 mm × 50 mm).

After completing the injection, the operator immediately withdrew the needle and observed the anesthetic effect. Throughout this process, strict aseptic techniques were adhered to, including wearing sterile gloves. A disposable acupotome measuring 1.0 mm × 50 mm was selected (Fig. 4) and inserted longitudinally at a 15° angle along the predetermined positioning point (Fig. 3C, D). Upon reaching the surface of the A1 pulley, fine longitudinal cutting operations were performed from proximal to distal 3 to 5 times to effectively release the thickened A1 annular ligament and restore its normal structure (Fig. 2D). During the procedure, the cutting direction was strictly aligned with the tendon orientation to prevent tendon injury, and the path was carefully maintained along the midline of the finger to avoid bilateral proper nerves and blood vessels, ensuring the safety of the operation.

Upon completion of the acupotomy release, the operator retracted the needle into the subcutaneous area and guided the patient to perform active flexion and extension of the affected finger to evaluate the therapeutic effect (Fig. 2E, F). The procedure continued until the original symptoms of snapping and compression disappeared completely, and the affected finger regained smooth and free motor function, indicating that the trigger finger symptoms had been effectively alleviated.

Following the completion of the procedure, the acupotome was removed, pressure was applied to stop any bleeding, and the affected finger was properly bandaged with sterile dressing to ensure that the wound remained clean and dry for 24 hours post-operation.

## 6. Results

### 6.1. VAS score

The Visual Analog Scale (VAS) was used, with a range of 0 to 10 points: 0 indicated no pain; scores below 3 indicated mild pain; scores of 4 to 6 indicated moderate pain; and scores of 7 to 10 indicated severe pain.

Before treatment, the patient’s VAS pain score was 8 (indicating severe pain). After treatment, the pain was significantly relieved, with a VAS score of 3 (indicating mild pain).

### 6.2. Range of motion (ROM)

Before treatment, the flexion range of motion (ROM) of the metacarpophalangeal joint of the thumb was <5°. After needle-knife treatment, the flexion ROM improved to nearly 90°.

### 6.3. *Quinnell grading system*^[6]^

The Quinnell grading system is utilized to assess the severity of trigger finger (stenosing tenosynovitis), which is divided into 5 grades. The specific criteria are as follows:

• Grade I: Free movement of the finger without pain (1 point).
• Grade II: Normal finger movement with mild pain (2 points).
• Grade III: Poor flexion and extension of the fingers, accompanied by noticeable resistance (3 points).
• Grade IV: Intermittent finger locking, requiring external force to complete flexion and extension movements (4 points).
• Grade V: Fingers in a locked and fixed state, mostly in unnatural flexion or extension positions, requiring external assistance for passive movement (5 points).

Before treatment, the patient had a Quinnell grade IV. After treatment, the patient’s condition improved to Quinnell grade I.

## 7. Discussion

Stenosing tenosynovitis of the thumb is more prevalent among middle-aged and elderly women. In severe cases, active flexion and extension of the thumb may become limited, requiring external assistance for movement, which can significantly impair patients’ daily life and work. This condition represents the most common form of stenosing tenosynovitis affecting the flexor tendon. Its prevalence may be attributed to prolonged gripping and repetitive thumb movements, leading to excessive friction on the tendon and degenerative changes in the anatomical structure.^[16]^

At the onset of the disease, patients often experience pain, stiffness, and other discomfort in the thumb, which typically improves after activity. For Quinnell Grade I patients and those with mild early symptoms, the thumb tendon exhibits no significant deformation, swelling, or only mild audible snapping without functional impairment. Conservative interventions – including activity modification, manual therapy, tendon gliding exercises, splinting, extracorporeal shock wave therapy, and therapeutic heat – effectively alleviate pain with favorable clinical outcomes and high patient compliance. Manual Therapy involves targeted mobilization of flexor tendons and forearm muscles to reduce pain and contractures.^[9]^ Tendon Gliding Exercises employ active tendon gliding and passive stretching of flexor tendons/joint capsules to prevent capsular shortening, enhance synovial fluid circulation, remodel scar tissue, and improve vascular/lymphatic drainage in congested tissues.^[8]^ Both manual therapy and tendon gliding exercises reduce adhesions, alleviate pain, and improve overall hand function,^[9]^ though neither achieves complete adhesion release and both require prolonged treatment durations.^[8]^ Splinting (e.g., MCP joint-blocking orthoses) limits digital motion to reduce mechanical stress on the A1 pulley, typically requiring 3 to 6 weeks of wear.^[17]^ Extracorporeal shock wave therapy primarily targets tendinopathic regions by reducing local inflammation and promoting neovascularization to accelerate tendon healing.^[18]^ Therapeutic Heat enhances blood flow to resolve edema, improves collagen extensibility/elasticity, and reduces stiffness/pain. Superficial modalities (2–3 cm depth: hot packs, paraffin wax) differ from deep modalities (5 cm depth: Ultrasound, diathermy) in tissue penetration.^[19]^

For patients with Quinnell Grade II and above, ultrasound imaging reveals that the inflamed tendon appears fusiform or elliptical.^[4]^ Stenosing tenosynovitis of the flexor pollicis longus tendon almost invariably occurs at the A1 pulley proximal to the metacarpophalangeal joint. Frequent flexion and extension activities, along with prolonged gripping, lead to local thickening and narrowing of the A1 pulley, obstructing the smooth gliding of the flexor pollicis longus tendon. This results in pain and limited thumb movement.

Topical steroid injections are commonly used as the first-line treatment, but they carry several potential complications. Blind injection techniques may inadvertently injure the tendon.^[4]^ While steroid injections can alleviate local pain, they do not address the issues of clicking and flexion limitation. Potential complications associated with topical steroid injections include infection, skin discoloration, tendon rupture, and lipoatrophy.^[20]^ Additionally, multiple injections at a specific site increase the risk of tendon rupture.^[21]^

Surgical intervention can rapidly relieve symptoms and is suitable for patients with tenosynovitis who have unsatisfactory outcomes from conservative treatments or experience severe recurrent symptoms. However, surgery involves significant tissue damage, a longer recovery period, higher costs, and the risk of postoperative scar adhesion leading to local stenosis.

Acupotomy not only achieves the purpose of releasing the narrowed A1 pulley through cutting but also significantly enhances the accuracy of ultrasound-guided acupotomology treatment. The specific advantages are as follows:

1. Ultrasound monitoring allows for passive flexion and extension of the thumb, enabling detection of the passage of the flexor pollicis longus tendon through the narrowed tendon sheath and precise determination of the A1 pulley’s position.
2. High-frequency ultrasound can visualize the thickening of the A1 pulley at the metacarpal head and the obstructed gliding of the flexor pollicis longus tendon within the tendon sheath.
3. Real-time observation of the anesthetic injection process and the direction of the needle-knife in soft tissues ensures precise administration.^[11]^
4. It enables evaluation of whether the tendon release is complete.

During local anesthesia guided by ultrasound, the tip of the syringe needle can be directly punctured to the surface of the A1 pulley for drug injection. The local diffusion of the drugs helps separate the tendon sheath from surrounding tissues, facilitating acupotomy operations. However, traditional acupotomy^[22]^ and percutaneous release^[23]^ techniques involve vertical needle insertion, which limits the longitudinal cutting range. Consequently, a single operation may not completely cut the thickened A1 pulley. Repeated needle insertions can impose significant psychological burden on patients and increase the risk of injury to tendons, blood vessels, and nerves if the needle is inserted too deeply or misplaced.

In this context, ultrasound-guided acupotomy was used to cut and release the A1 pulley along the surface of the flexor pollicis longus tendon. This approach reduces the risk of tendon injury, and the completeness of the release can be assessed by dynamically observing the tendon’s sliding within the tendon sheath. The procedure can be completed in approximately 5 minutes in an outpatient setting, resolving the issue with a single treatment. It is simple, safe, and effective.

In conclusion,ultrasound-guided acupotomy release for treating thumb stenosing tenosynovitis offers precise localization of the stenotic tendon sheath, preoperative evaluation of tendon gliding obstruction, and real-time monitoring of needle insertion and release during the procedure. This method is both effective and safe.

## Author contributions

**Data curation:** Jingxi Ou, Chengcheng Liu.

**Conceptualization:** Chengcheng Liu, Li Liu, Pengyan Han.

**Funding acquisition:** Li Liu.

**Investigation:** Chengcheng Liu.

**Methodology:** Chengcheng Liu, Li Liu, Pengyan Han.

**Supervision:** Chengcheng Liu, Li Liu.

**Writing – original draft:** Jingxi Ou.

**Writing – review & editing:** Jingxi Ou, Chengcheng Liu.

## References

[1] Wong AL Wong MJ Parker R Wheelock ME. Presentation and aetiology of paediatric trigger finger: a systematic review. J Hand Surg Eur Vol. 2022;47:192–6. 34610771 10.1177/17531934211035642 PMC8873964

[2] Sood RF Westenberg RF Winograd JM Eberlin KR Chen NC. Genetic risk of trigger finger: results of a genomewide association study. Plast Reconstr Surg. 2020;146:165e–76e. 10.1097/PRS.0000000000006982 32740585

[3] Lin Y-C Shieh S-J. Extensor-pollicis-longus or -brevis tendon rupture after corticosteroid injection. Formosan J Surg. 2016;49:15–9.

[4] Bianchi S Gitto S Draghi F. Ultrasound features of trigger finger: review of the literature. J Ultrasound Med. 2019;38:3141–54. 31106876 10.1002/jum.15025

[5] Vasiliadis AV Itsiopoulos I. Trigger finger: an atraumatic medical phenomenon. J Hand Surg Asian Pac Vol. 2017;22:188–93. 28506168 10.1142/S021881041750023X

[6] Aref HA Fatemeh S Hosein KM. Comparison between corticosteroid injection and surgery in the treatment of trigger finger. J Transl Intern Med. 2014;2:132–5.

[7] Leow MQH Hay ASR Ng SL. A randomized controlled trial comparing ketorolac and triamcinolone injections in adults with trigger digits. J Hand Surg Eur Vol. 2018;43:936–41. 29448917 10.1177/1753193418756808

[8] Donati D Ricci V Boccolari P. Trigger finger: a narrative review of dynamic ultrasound and personalized therapies [published online ahead of print March 21, 2025]. J Clin Ultrasound. doi: 10.1002/jcu.23971. 10.1002/jcu.23971 40119539

[9] Donati D Ricci V Boccolari P. From diagnosis to rehabilitation of trigger finger: a narrative review. BMC Musculoskelet Disord. 2024;25:1061. 39716186 10.1186/s12891-024-08192-5 PMC11664832

[10] Taras JS Raphael JS Pan WT Movagharnia F Sotereanos DG. Corticosteroid injections for trigger digits: is intrasheath injection necessary? J Hand Surg Am. 1998;23:717–22. 9708388 10.1016/S0363-5023(98)80060-9

[11] Wang J Zhao J-G Liang C-C. Percutaneous release, open surgery, or corticosteroid injection, which is the best treatment method for trigger digits? Clin Orthop Relat Res. 2013;471:1879–86. 23208122 10.1007/s11999-012-2716-6 PMC3706641

[12] Bruijnzeel H Neuhaus V Fostvedt S Jupiter JB Mudgal CS Ring DC. Adverse events of open A1 pulley release for idiopathic trigger finger. J Hand Surg Am. 2012;37:1650–6. 22763058 10.1016/j.jhsa.2012.05.014

[13] Ryzewicz M Wolf JM. Trigger digits: principles, management, and complications. J Hand Surg Am. 2006;31:135–46. 16443118 10.1016/j.jhsa.2005.10.013

[14] Nakagawa H Redmond T Colberg R. Ultrasound-guided A1 pulley release: a systematic review. J Ultrasound Med. 2023;42:2491–9. 37401544 10.1002/jum.16294

[15] Yang J Ma B Zhong H Zhang Y Zhu J Ni Y. Ultrasound-guided percutaneous A1 pulley release by acupotomy (needle-knife): a cadaveric study of safety and efficacy. J Pain Res. 2022;15:413–22. 35173479 10.2147/JPR.S349869 PMC8842668

[16] Sbernardori MC Mazzarello V Tranquilli-Leali P. Scanning electron microscopic findings of the gliding surface of the A1 pulley in trigger fingers and thumbs. J Hand Surg Eur Vol. 2007;32:384–7. 17399869 10.1016/J.JHSB.2007.01.013

[17] Huisstede BMA Hoogvliet P Coert JH Fridén J; European HANDGUIDE Group. Multidisciplinary consensus guideline for managing trigger finger: results from the european HANDGUIDE study. Phys Ther. 2014;94:1421–33. 24810861 10.2522/ptj.20130135

[18] Tenforde AS Borgstrom HE DeLuca S. Best practices for extracorporeal shockwave therapy in musculoskeletal medicine: clinical application and training consideration. PM R. 2022;14:611–9. 35187851 10.1002/pmrj.12790 PMC9321712

[19] Ferrara PE Codazza S Maccauro G Zirio G Ferriero G Ronconi G. Physical therapies for the conservative treatment of the trigger finger: a narrative review. Orthop Rev (Pavia). 2020;12:8680. 32913608 10.4081/or.2020.8680 PMC7459363

[20] Nichols AW. Complications associated with the use of corticosteroids in the treatment of athletic injuries. Clin J Sport Med. 2005;15:E370. 10.1097/01.jsm.0000179233.17885.18 16162982

[21] Pfenninger JL. Injections of joints and soft tissue: part II. Guidelines for specific joints. Am Fam Physician. 1991;44:1690–701. 1950966

[22] Liang Y Chen L Cui Y Du C-X Xu Y-X Yin L-H. Ultrasound-guided acupotomy for trigger finger: a systematic review and meta-analysis. J Orthop Surg Res. 2023;18:678. 37705066 10.1186/s13018-023-04127-3 PMC10498646

[23] Marij Z Aurangzeb Q Rizwan HR Haroon R Pervaiz MH. Outpatient percutaneous release of trigger finger: a cost effective and safe procedure. Malays Orthop J. 2017;11:52–6. 28435575 10.5704/MOJ.1703.021 PMC5393115

